# A perspective on the need and current status of efficient sex separation methods for mosquito genetic control

**DOI:** 10.1186/s13071-018-3222-9

**Published:** 2018-12-24

**Authors:** Philippos Aris Papathanos, Kostas Bourtzis, Frederic Tripet, Hervé Bossin, Jair Fernandes Virginio, Margareth Lara Capurro, Michelle Cristine Pedrosa, Amadou Guindo, Lakamy Sylla, Mamadou B. Coulibaly, Franck Adama Yao, Patric Stephane Epopa, Abdoulaye Diabate

**Affiliations:** 1 Department of Entomology, The Robert H Smith Faculty of Agriculture, Food and Environment, Hebrew University of Jerusalem, Rehovot, Israel; 2 Insect Pest Control Laboratory, Joint FAO/IAEA Division of Nuclear Techniques in Food and Agriculture, Vienna, Austria; 3 Centre for Applied Entomology and Parasitology, School of Life Sciences, Keele University, Keele, Staffordshire UK; 4 Laboratoire d’Entomologie Médicale, Institut Louis Malardé, BP 30, 98713 Papeete, French Polynesia; 5 Aix Marseille Univ, IRD, AP-HM, SSA, VITROME, IHU-Méditerranée infection, Marseille, France; 6 Biofabrica Moscamed Brazil, Industrial District, Juazeiro, BA Brazil; 7 Department of Parasitology, Institute of Biomedical Sciences, University of São Paulo, São Paulo, SP Brazil; 8 Malaria Research and Training Center, Université des Sciences, des Techniques et des Technologies de Bamako, Point G, Bamako, BP: 1805, Mali; 9 Institut de Recherche en Sciences de la Sante, Centre Muraz, Bobo-Dioulasso, Burkina Faso

**Keywords:** Mosquito, Genetic control, Sex separation, Sexing, Genetic sexing strains, Sterile insect technique, Gene drive

## Abstract

Major efforts are currently underway to develop novel, complementary methods to combat mosquito-borne diseases. Mosquito genetic control strategies (GCSs) have become an increasingly important area of research on account of their species-specificity, track record in targeting agricultural insect pests, and their environmentally non-polluting nature. A number of programs targeting *Aedes* and *Anopheles* mosquitoes, vectors of human arboviruses and malaria respectively, are currently being developed or deployed in many parts of the world. Operationally implementing these technologies on a large scale however, beyond proof-of-concept pilot programs, is hampered by the absence of adequate sex separation methods. Sex separation eliminates females in the laboratory from male mosquitoes prior to release. Despite the need for sex separation for the control of mosquitoes, there have been limited efforts in recent years in developing systems that are fit-for-purpose. In this special issue of Parasites and Vectors we report on the progress of the global Coordinated Research Program on “Exploring genetic, molecular, mechanical and behavioural methods for sex separation in mosquitoes” that is led by the Insect Pest Control Subprogramme of the Joint FAO/IAEA Division of Nuclear Techniques in Food and Agriculture with the specific aim of building efficient sex separation systems for mosquito species. In an effort to overcome current barriers we briefly highlight what we believe are the three main reasons why progress has been so slow in developing appropriate sex separation systems: the availability of methods that are not scalable, the difficulty of building the ideal genetic systems and, finally, the lack of research efforts in this area.

## Background

Mosquito genetic control strategies (GCSs) come in a wide variety of flavors (see for example [1]). By being mating-based, GCSs are extremely species-specific, and benefit from the self-dispersal and mate-seeking behavior of released male individuals. There are two main characteristics that are useful in distinguishing the mosquito GCSs currently being developed: the desired outcome of the program: population suppression vs replacement; and the persistence or invasiveness of the “agent” that is released - self-limiting *versus* self-sustaining. While both the underlying genetic basis and the scale of the release is strategy-dependent, all GCSs have the prerequisite for implementation that only males should be released in the field - with the exceptions of population replacement using maternally inherited symbionts [2, 3] and the Trojan Female Technique [4, 5]. Female mosquitoes need to be removed prior to release for multiple reasons: First and foremost, released females can themselves contribute towards nuisance and disease transmission. Secondly, released females may inhibit the dispersal and mating rate of released males to wild females, reducing the efficiency of the releases [6–8]. Thirdly, rearing females that have to be discarded prior to release is, essentially, a logistical waste on facility resources halving the rearing capacity of any facility. Finally, the release of females can under certain circumstances, even compromise the long-term sustainability of programs, most notably those employing the Incompatible Insect Technique (IIT) [9–13]. IIT relies on sustained releases of *Wolbachia*-infected, incompatible males to sterilize the targeted female population. If the *Wolbachia* symbiont becomes established in the wild population through the accidental release of fertile females, this could result in the loss of the genetic incompatibility between the laboratory strain and the wild population. Importantly, as the population is suppressed, the risk of field establishment of the symbiont increases significantly per (accidentally) released female as the total number of wild females is comparatively low.

Thus, the ability to separate males from females in the laboratory setting at a large scale is of critical importance for the broad implementation of mosquito GCSs. However, a system that allows high-throughput, reliable sex separation does not exist for any mosquito species that meets the criteria needed for mass-release programs. Indeed, since our last technology review almost a decade ago [14] there has been no new system developed, even at the proof-of-concept stage, for any mosquito beyond what was available then. A coordinated research project entitled “Exploring genetic, molecular, mechanical and behavioural methods of sex separation in mosquitoes” was therefore launched in 2013 and was coordinated by the Insect Pest Control Sub-programme of the Joint Division of Nuclear Techniques in Food and Agriculture of the Food and Agriculture Organization of the United Nations (FAO) and the International Atomic Energy Agency (IAEA) with the goal to explore classical genetic, molecular, mechanical, behavioral, developmental and symbiont-based approaches for sex separation in mosquitoes. Over the last five years this consortium has made several important steps towards building the essential components needed for the development of novel sex separation methods or towards improving established methods. To ensure success and finally close this technological gap we believe it may be useful to address possible reasons of why this gap still exists today.

### Currently available techniques are not scalable

Basic sex separation methods are available for some mosquito species based on naturally-occurring differences between male and female mosquitoes. These methods (described below) have enabled programs to proceed to limited-scale pilot releases of sterile (SIT) and/or incompatible (IIT) males, which would have not been possible were it not for these sex separation methods. These releases have been, and will continue to be, necessary in demonstrating proof-of-principle of the GCSs being developed and for collecting necessary data on program logistics, acceptability and the performance of released individuals, e.g. dispersal distance, mating frequency and population suppression. Nevertheless, these rudimentary sex separation methods are ultimately not scalable for mass-release programs without further improvement, assuming a continuous operational target of 100% male-only releases. To do this is prohibitively expensive to scale-up because these (i) rely on manual sorting by expert technicians, (ii) sorting occurs at later stages of mosquito development (pupae or adult) decreasing facility efficiency, (iii) most methods inflict significant damage reducing insect quality. Moreover, the penetrance and degree of dimorphisms of these naturally-occurring traits can vary dramatically between species and strains and/or rearing conditions. Unfortunately, it is likely that their availability has resulted in some degree complacency in the investment of research efforts in building improved systems, effectively pushing the ball down the road while other operational issues are prioritized. In the following paragraphs we provide a current status of these available sex separation methods.

### Size and developmental dimorphisms

*Aedes* and *Culex* and some *Anopheles* mosquito species display significant size differences at the pupal stage; females are larger than male pupae [15]. Male mosquitoes also develop faster than females, a widely occurring phenomenon in insects called protandry. Several programs have taken advantage of these two naturally-occurring traits by removing larger pupae on the first days of synchronous pupation. For example, in an IIT trial in the Pacific atoll of Tetiaroa (French Polynesia), standardized rearing of *Aedes polynesiensis* allowed for low female percentage (0.01 %) during one year of continuous production and release (H. Bossin in preparation). In an ongoing pilot trial in Singapore implementing the IIT or the SIT/IIT to target *Ae. aegypti* and using the current standards based on size separation and protandry was able to achieve sex separation with a low percentage of females at 0.1-0.02% (Lee Ching Ng and Cheong Huat Tan, personal communication). In the Brazil RIDL pilot programs, sex separation using these approaches resulted in 0.02 ± 0.004 % females at an average loss of 19.3 % males [16, 17]. Laboratory workers with extensive training in the sorting process, were able to sex separate from rearing trays containing 9000 individuals (larvae and pupa) in an average time of 5’ ± 0.17’ with low initial female percentage (0.51 % ± 0.06 %) [16]. Importantly, in most *Anophelines*, there is no significant difference in pupal size between males and females and relying on protandry alone is not sufficient.

### Morphological sex separation

In many important mosquito species, sex is indistinguishable during their embryonic and early larval stages. At the pupal stage, males and females can be separated under the microscope based on morphological differences in the terminal abdominal segments. Laboratory workers can be trained to separate males from females. This in fact is a routine laboratory procedure for setting up crosses and to collect virgin females. Skilled staff can sex pupae at a rate of approximately 500 pupae per hour with an error rate between 0.05-1 % (numbers from various programs that authors are involved in). Therefore, human resources are the limiting factor for mass production of males. Unfortunately, for *Anopheles* mosquitoes, this method is the only one available to separate sexes prior to the more fragile adult stage. Errors made during pupae sexing can be corrected through visual inspection of recently emerged adults and removal of accidental females, a step that requires further staff time. Aside from being time consuming, this method can also cause significant amounts of mortality in pupae.

### Behavioral differences

For blood-feeding mosquitoes, methods to separate the sexes at the adult stage have been developed that take advantage of female-specific blood feeding. Several studies have evaluated the spiking of blood meals with insecticides or other insect toxins and have reported high rates of female elimination. For example, using malathion led to 95 % female elimination [18]*.* In some studies, however, treatment resulted in significant losses of males due to inadvertent exposure. As a result, proposals have also been put forward to take advantage of female host-seeking behavior, by creating systems that could capture females drawn to host cues [15]. Male quality is of concern when using this approach because adults are the most fragile developmental stage, because of inadvertent male exposure to the toxins, and because of the long adult holding time required until an effect is observed. Finally, the economics of rearing females till the adult stage is also a significant barrier that must be overcome.

### Genetic sexing strains are ideal**,** but difficult to generate

Of all possible methods to separate insect sexes in a mass-rearing setting, a genetic sexing strain (GSS) is the ideal system. GSSs are laboratory strains of the target insect whose genetics have been manipulated to allow efficient sex separation. Typically, GSSs are made by first using classical mutagenesis to create and select a marker, for example cuticle color, chemical or temperature sensitivity. In a second step the allele that provides the positive selection is moved then to the Y chromosome to link it to maleness [19]. GSSs have been generated in dozens of insect species, including mosquitoes. In the malaria mosquitoes *Anopheles albimanus* [20] and *Anopheles arabiensis* [21], GSSs were developed using translocations of insecticide resistance alleles to the Y chromosome. The *An. arabiensis* strain was based on Y-chromosome linkage of a mutation at the *rdl* locus making males insensitivity to dieldrin treatment. This system did not progress further in part because of environmental concerns with the use of this insecticide, because dieldrin based male selection at the embryonic stage was not very effective, and finally because the strain suffered from low productivity [21]. The *An. albimanus* strain (aptly named *macho*) was successfully used in the El Salvador trial but the program was stopped and the strain lost. Of the dozen or so species in which GSSs were developed, the strains of agricultural fruit fly pest *Ceratitis capitata* developed by the Joint FAO/IAEA Insect Pest Control Laboratory in Seibersdorf have been the most successful, and have in fact enabled the currently operational worldwide SIT programs. The *C. capitata* GSS currently in use is based on two mutations, the temperature-sensitive lethal (*tsl*) that is used to eliminate females [22] and the closely linked white pupae (*wp*) [23], which acts as a secondary visible marker. Females homozygous for *tsl* are sensitive to high temperatures, while males that are *tsl*+ hemizygous on the Y are insensitive. Sexing is achieved by incubating eggs at 34 ^o^C for 24 hours killing almost 100 % of female embryos, even in large facilities where hundreds of millions of eggs are treated every day [19]. The *wp* color marker, with white females versus brown males, is used at the pupal stage to quality control the efficiency of female elimination in each egg batch. The *tsl/wp* mutations display a number of important properties that make system so optimal for operation use: (i) treatment of embryos is very simple, accurate and cheap to implement; (ii) eliminating females at this stage minimizes rearing costs; (iii) the penetrance of the tsl phenotype is inducible (through temperature) but otherwise does not have a significant negative impact on the maintenance of the colony which is true-breeding and relatively simple to maintain (although all facilities using this strain require the use of a small “Filter colony” that is maintained under relaxed rearing conditions and is used to remove recombinants to ensure integrity of the GSS [24]); (iv) male quality is not affected by the temperature treatment, and the 50 % fertility cost resulting from the translocation through gamete imbalance can be overcome by loading production cages (those yielding populations for release) with an excess of females; (v) tsl homozygous females develop more slowly than males that are hemizygous for *tsl*+ on the Y, even without heat treatment. The first day of pupal collection yields virtually only male pupae, but the collection on the 5th day contains mostly females. These dimorphic developmental rates provides an indicator of the larval rearing conditions (poor rearing conditions diminish developmental dimorphisms) and a method to bias the production cages with an excess of females without the need of a pupal separator; (vi) finally, *tsl* mutant females accidentally escaping from the facility are unlikely to survive in the natural environment because susceptibility to high temperatures affects all developmental stages [19].

Developing GSSs *de novo* in mosquito disease vectors or even closely related agricultural pests have proven very difficult for a host of reasons, but mainly because generating suitable mutations with classical genetics is a very unpredictable and fortuitous process. To give an example, Mullins & Rubin conducted an F1 non-complementation screen of 36,000 mutagenized autosomes and recovered only two temperature sensitive alleles of the *sevenless* gene of *Drosophila melanogaster* [25]. Running a screen of a similar magnitude, with the aim of targeting a single target gene in a mosquito species would be prohibitively difficult due to inherent limitations and logistics of performing genetic crosses and maintaining individual mosquito strains. There are roughly 100 temperature sensitive loci known in *Drosophila* and so if similar numbers exist in the mosquito, following such an approach may be feasible. Indeed, this has been done previously in one *Culex* species [26]. However, even if a suitable mutant were identified, translocating the selectable marker to the Y chromosome as a second step would also be very time-consuming, labor-intensive, unpredictable and the fertility and viability of males harboring translocated Ys can vary dramatically. This is especially the case with *Aedes* species that do not have heteromorphic sex chromosomes, but instead contain the male-determining gene Nix in a small, non-recombining M locus on chromosome 1 [27]. Finally, the expertise and knowledge needed to perform such classical genetic approaches for generating GSSs, at least in mosquitoes, have become a disappearing art form with the arrival of the molecular biology era and its promise to precisely engineer alternatives. These hopes have been followed by some progress, for example with the development of transgenic fluorescent markers expressed either from male-specific regulatory elements or placed directly on the Y chromosome [28, 29]. Other strategies focused on developing negative selection systems, for example lethal transgenes expressed using female-specific promoters or splicing [30]. However, these very promising systems have thus far only been tested on a very small scale, and many important questions, e.g. scalability, stability, sexing accuracy, strain productivity, costs etc., cannot be addressed precisely. Perhaps more importantly, GSSs built using transgenic technologies are not being readily adopted by programs that don't inherently rely on transgenes for generating the primary control trait (e.g. sterility with SIT or IIT) given the current public opposition to the use and release of transgenic organisms.

### Biased research focus on developing the primary components of genetic control

The last 10 years have seen unprecedented progress in the application and development of the primary genetic components of mosquito control strategies. Here, we define primary as the actual genetic basis for control, e.g. sterility (SIT), incompatibility (IIT), female-elimination (RIDL). However, there has not been similar progress or investment in developing many of the secondary technologies that are critical to their success, including sex separation. A contributing factor to this has undoubtedly been the prioritization of work that exudes cutting-edge novelty, like engineering gene drive or under-dominance, because these can be “sold” as more innovative and as dramatic improvements to status quo methods. To give a single example, the first paper reporting gene drive in an *Anopheles* [31] was published in the journal Nature, even though the gene we targeted in that work was a fluorescent transgene that contained the recognition sequence of the naturally-occurring endonuclease conferring no phenotype useful for control purposes. Essentially, this was proof-of-principle of the feasibility of the approach and the proposed technology was then, and remains today, decades away from field implementation. By comparison, a paper that reported on the first use of dsRNA for sex separation of *Aedes* larvae, which could in theory be directly applied to a large number of GCSs immediately, was published in a much lower impact-factor journal [32]. It is therefore not surprising that the vast majority of laboratories seeking to work in state-of-the-art insect genetics are drawn to developing novel methods of genetic control, rather than focusing on contributing to the incremental steps needed in realizing these. In a period when a proposals’ “novelty and innovativeness” are so highly ranked in the selection of projects to support financially, how can a proposal seeking to develop, for example genetic sexing strains using classical genetic approaches, appear competitive? Clearly, to support the development of such technology will require directed calls by agencies committed to the implementation of genetic control of mosquitoes. From the researchers’ side, it is important to realize that currently available technologies like SIT or IIT (or their combination) are now ready and suitable for large-scale implementation. The challenge of building novel self-sustaining GCSs or population replacement is a worthwhile goal for all the promises it holds, but should this occur at the expense of implementing those currently available?

### Future perspectives

There is an increasing need to develop highly efficient sex separation methods for mosquitoes. Progress here will have to begin by the community and its funders accepting that current approaches, e.g. pupal size or protandry, will not be able to meet the needs imposed on sex separation by large-scale programs without significant further improvements to their underlying systems. There are ongoing efforts to increase female elimination levels based on either size dimorphism or protandry by incorporating automated mechanical methods for size separation or genetics. For example, M. Zacarés and colleagues have reported in this issue on the development of an automated pupal size estimator developed by Grupo Tragsa and tested with laboratory samples of *Aedes aegypti*, *Ae. albopictus* and *Ae. polynesiensis*. Their data are very encouraging showing that enhanced sexual size dimorphism (SSD)-based sex sorting methods can be applied to mosquito mass-rearing facilities to efficiently produce batches of male-only pupae with a male recovery up to 99% and minimal female contamination which can be even under 0.1% [33]. For programs including an IIT component, a beneficial side-effect of mosquito infection with the *Wolbachia w*Pip strain is enhanced protandry [34, 35]. Furthermore, since protandry is in part genetically determined, selection of increased differences in adult eclosion in *Ae. albopictus* has also led to the generation of improved strains showing higher discrimination [36].

At the same time, many of the technological advances and knowledge needed to build efficient GSSs are now at our disposal. Insect genome sequencing and comparative genomics have made it more likely that candidate target genes can be identified in any mosquito. For example, there are ongoing efforts to identify the loci responsible for the *tsl* mutation [22] in *C. capitata*, by comparative analysis of GSSs carrying translocations and/or inversions and reference wild type strains and comparative transcriptomic analysis of mutant and wild type flies. This work may reveal the nature of these mutants, and make it possible to target orthologs in the mosquito to engineer similar temperature sensitive loci. Genome engineering methods are now also available such as the CRISPR/Cas system for the targeted mutagenesis of any newly discovered target genes. One of the advantages provided by CRISPR/Cas engineering is the introduction of specific changes that lead to the desired mutant phenotypes without the need to insert exogenous DNA. As a result, it may be possible that in the future, strains generated through targeted mutagenesis may not be classified as genetically modified organisms and may thus have a simpler path to acceptance and application, although it is currently not clear whether this will be the case. Importantly, there has been significant progress in mosquito engineering using CRISPR [37–43]. For example, genes, whose enzymatic activity results in wild type cuticle or eye color that within a GSS strain can act as a secondary visible marker have already been mutated and characterized in several mosquito species [44]. Y-chromosome or M locus-specific sequences have also been characterized in several major disease vectors [45, 46], making it possible to specifically insert within them the rescue alleles for male selection. These tools have also enabled studies of mosquito Y chromosome biology and have led to the identification of the primary signals, the so-called M-factors, which define and orchestrate male sex determination. Mis-expression of these M-factors (Yob [47], Nix [27], Guy1 [48]) in females is now being explored as a method to either induce female-specific lethality or to phenotypically convert genetic females into males. The former would occur if the M-factor is also involved in dosage compensation, as it appears to be in *Anopheles gambiae* [49], while the latter is more likely in *Aedes* species that have an autosomal M-factor and homomorphic sex chromosomes. To take advantage of these M-factors for sex separation will require early embryonic repressible expression systems, variants of which are already available in mosquitoes using transgenic constructs such as the tet-off system [30, 50].

## Conclusions

Despite the progress achieved during the last few years, there is still a need for the development of efficient and robust sex separation methods, ideally based on genetic sexing strains, for *Anopheles* and *Aedes* mosquitoes as well as for other major insect pests and disease vector species. Recognizing this need, the Insect Pest Control Subprogramme of the Joint FAO/IAEA Division of Nuclear Techniques in Food and Agriculture is planning for a new Coordinated Research Project on “Generic approach for the development of genetic sexing strains for SIT applications”. Commitments like these both from researchers and funding agencies are now urgently needed. With sustained or increased interest, funding, research and coordinated development, we remain optimistic that efficient sexing systems can be developed for mosquitoes, and these will inevitably enable large-scale implementation of both available GCSs and those in the horizon.

## Abbreviations

GCS: Genetic Control Strategy
GSS: Genetic Sexing Strain
IIT: Incompatible Insect Technique
SIT: Sterile Insect Technique

## References

[1] Macias VM, Ohm JR, Rasgon JL. Gene drive for mosquito control: where did it come from and where are we headed? Int J Environ Res Public Health. 2017;14 10.3390/ijerph14091006. 10.3390/ijerph14091006 PMC5615543 28869513

[2] Moreira LA, Iturbe-Ormaetxe I, Jeffery JA, Lu G, Pyke AT, Hedges LM (2009). A *Wolbachia* symbiont in *Aedes aegypti l*imits infection with dengue, chikungunya, and *Plasmodium*. Cell..

[3] Hoffmann AA, Montgomery BL, Popovici J, Iturbe-Ormaetxe I, Johnson PH, Muzzi F (2011). Successful establishment of *Wolbachia* in *Aedes* populations to suppress dengue transmission. Nature..

[4] Gemmell NJ, Jalilzadeh A, Didham RK, Soboleva T, Tompkins DM (2013). The Trojan female technique: a novel, effective and humane approach for pest population control. Proc Biol Sci..

[5] Dowling DK, Tompkins DM, Gemmell NJ (2015). The Trojan Female Technique for pest control: a candidate mitochondrial mutation confers low male fertility across diverse nuclear backgrounds in *Drosophila melanogaster*. Evol Appl..

[6] Orozco D, Hernández MR, Meza JS, Quintero JL (2013). Do sterile females affect the sexual performance of sterile males of *Anastrepha ludens* (Diptera: Tephritidae)?. J Appl Entomol..

[7] Flores S, Montoya P, Toledo J (2014). Estimation of populations and sterility induction in *Anastrepha ludens* (Diptera: Tephritidae) fruit flies. J Econ..

[8] Vreysen MJB, Barclay HJ, Hendrichs J (2014). Modeling of preferential mating in areawide control programs that integrate the release of strains of sterile males only or both sexes. Ann Entomol Soc Am..

[9] Laven H (1967). Eradication of *Culex pipiens fatigans* through cytoplasmic incompatibility. Nature..

[10] Calvitti M, Moretti R, Lampazzi E, Bellini R, Dobson SL (2010). Characterization of a new Aedes *albopictus* (Diptera: Culicidae)-*Wolbachia pipientis* (Rickettsiales: *Rickettsiaceae*) symbiotic association generated by artificial transfer of the wPip strain from *Culex pipiens* (Diptera: Culicidae). J Med Entomol..

[11] Chen L, Zhu C, Zhang D (2013). Naturally occurring incompatibilities between different *Culex pipiens pallens* populations as the basis of potential mosquito control measures. PLoS Negl Trop Dis..

[12] Bian G, Joshi D, Dong Y, Lu P, Zhou G, Pan X (2013). *Wolbachia* invades *Anopheles stephensi* populations and induces refractoriness to *Plasmodium* infection. Science..

[13] Zabalou S, Riegler M, Theodorakopoulou M, Stauffer C, Savakis C, Bourtzis K (2004). *Wolbachia*-induced cytoplasmic incompatibility as a means for insect pest population control. Proc Natl Acad Sci USA..

[14] Papathanos PA, Bossin HC, Benedict MQ, Catteruccia F, Malcolm CA, Alphey L (2009). Sex separation strategies: past experience and new approaches. Malar J.

[15] Gilles JRL, Schetelig MF, Scolari F, Marec F, Capurro ML, Franz G (2014). Towards mosquito sterile insect technique programmes: exploring genetic, molecular, mechanical and behavioural methods of sex separation in mosquitoes. Acta Trop..

[16] Carvalho DO, Nimmo D, Naish N, McKemey AR, Gray P, Wilke ABB, et al. Mass production of genetically modified *Aedes aegypti* for field releases in Brazil. J Vis Exp. 2014;(83):e3579. 10.3791/3579. 10.3791/3579 PMC4063546 24430003

[17] Garziera L, Pedrosa MC, de Souza FA, Gómez M, Moreira MB, Virginio JF (2017). Effect of interruption of over-flooding releases of transgenic mosquitoes over wild population of *Aedes aegypti*: two case studies in Brazil. Entomol Exp Appl..

[18] Lowe RE (1981). Fowler J, Bailey DL, Dame DA, Savage KE, Others. Separation of sexes of adult *Anopheles albimanus* by feeding of insecticide-laden blood. Mosq News..

[19] Franz G (2005). Genetic sexing strains in Mediterranean fruit fly, an example for other species amenable to large-scale rearing for the sterile insect technique. Sterile Insect Technique.

[20] Kaiser PE, Seawright JA, Dame DA, Joslyn DJ (1978). Development of a genetic sexing system for *Anopheles albimanus*. J Econ Entomol..

[21] Yamada H, Vreysen MJB, Bourtzis K, Tschirk W, Chadee DD, Gilles JRL (2015). The *Anopheles arabiensis* genetic sexing strain ANO IPCL1 and its application potential for the sterile insect technique in integrated vector management programmes. Acta Trop..

[22] Franz G, Gencheva E, Kerremans P (1994). Improved stability of genetic sex-separation strains for the Mediterranean fruit fly, *Ceratitis capitata*. Genome..

[23] Rössler Y (1979). The genetics of the Mediterranean fruit fly: a “White Pupae” mutant. Ann Entomol Soc Am..

[24] Fisher K, Caceres C, et al. A filter rearing system for mass reared genetic sexing strains of Mediterranean fruit fly (Diptera: Tephritidae). Area-wide control of fruit flies and other insect pests Joint proceedings of the international conference on area-wide control of insect pests, 28 May-2 June, 1998 and the Fifth International Symposium on Fruit Flies of Economic Importance, Penang, Malaysia, 1-5 June, 1998. Pulau Pinang: Penerbit Universiti Sains Malaysia; 2000. p. 543–50.

[25] Mullins MC, Rubin GM (1991). Isolation of temperature-sensitive mutations of the tyrosine kinase receptor sevenless (sev) in *Drosophila* and their use in determining its time of action. Proc Natl Acad Sci USA..

[26] Sakai RK, Baker RH (1974). Others. Induction of heat-sensitive lethals in Culex tritaeniorhynchus by ethyl methanesulfonate. Mosq News..

[27] Hall AB, Basu S, Jiang X, Qi Y, Timoshevskiy VA, Biedler JK (2015). Sex determination. A male-determining factor in the mosquito *Aedes aegypti*. Science..

[28] Bernardini F, Galizi R, Menichelli M, Papathanos P-A, Dritsou V, Marois E (2014). Site-specific genetic engineering of the *Anopheles gambiae* Y chromosome. Proc Natl Acad Sci USA..

[29] Catteruccia F, Benton JP, Crisanti A (2005). An *Anopheles* transgenic sexing strain for vector control. Nat Biotechnol..

[30] Fu G, Lees RS, Nimmo D, Aw D, Jin L, Gray P (2010). Female-specific flightless phenotype for mosquito control. Proc Natl Acad Sci USA..

[31] Windbichler N, Menichelli M, Papathanos PA, Thyme SB, Li H, Ulge UY (2011). A synthetic homing endonuclease-based gene drive system in the human malaria mosquito. Nature..

[32] Whyard S, Erdelyan CNG, Partridge AL, Singh AD, Beebe NW, Capina R (2015). Silencing the buzz: a new approach to population suppression of mosquitoes by feeding larvae double-stranded RNAs. Parasit Vectors..

[33] Zacarés M, Salvador-Herranz G, Almenar D, Tur C, Argilés R, Bourtzis K, Bossin H, Pla I. Exploring the potential of computer vision analysis of pupae size dimorphism for adaptive sex sorting systems of various vector mosquito species. Parasit Vectors (this issue). 10.1186/s13071-018-3221-x. 10.1186/s13071-018-3221-x PMC6304766 30583722

[34] Zhang D, Zheng X, Xi Z, Bourtzis K, Gilles JRL (2015). Combining the sterile insect technique with the incompatible insect technique: I-impact of *Wolbachia* infection on the fitness of triple- and double-infected strains of *Aedes albopictus*. PLoS One..

[35] Puggioli A, Calvitti M, Moretti R, Bellini R (2016). wPip *Wolbachia* contribution to *Aedes albopictus* SIT performance: advantages under intensive rearing. Acta Trop..

[36] Bellini R, Puggioli A, Balestrino F, Carrieri M, Urbanelli S. Exploring protandry and pupal size selection for *Aedes albopictus* sex separation. Parasit Vectors. (this issue). 10.1186/s13071-018-3213-x. 10.1186/s13071-018-3213-x PMC6304755 30583737

[37] Li M, Akbari OS, White BJ (2018). Highly efficient site-specific mutagenesis in malaria mosquitoes using CRISPR. G3.

[38] Dong Y, Simões ML, Marois E, Dimopoulos G (2018). CRISPR/Cas9 -mediated gene knockout of *Anopheles gambiae* FREP1 suppresses malaria parasite infection. PLoS Pathog..

[39] Vinauger C, Lahondère C, Wolff GH, Locke LT, Liaw JE, Parrish JZ (2018). Modulation of host learning in *Aedes aegypti* mosquitoes. Curr Biol.

[40] Galizi R, Hammond A, Kyrou K, Taxiarchi C, Bernardini F, O’Loughlin SM (2016). A CRISPR-Cas9 sex-ratio distortion system for genetic control. Sci Rep..

[41] Zhang Y, Zhao B, Roy S, Saha TT, Kokoza VA, Li M (2016). microRNA-309 targets the Homeobox gene SIX4 and controls ovarian development in the mosquito *Aedes aegypti*. Proc Natl Acad Sci USA..

[42] Itokawa K, Komagata O, Kasai S, Ogawa K, Tomita T (2016). Testing the causality between CYP9M10 and pyrethroid resistance using the TALEN and CRISPR/Cas9 technologies. Sci Rep..

[43] Kistler KE, Vosshall LB, Matthews BJ (2015). Genome engineering with CRISPR-Cas9 in the mosquito *Aedes aegypti*. Cell Rep..

[44] Li M, Bui M, Yang T, Bowman CS, White BJ, Akbari OS (2017). Germline Cas9 expression yields highly efficient genome engineering in a major worldwide disease vector, *Aedes aegypti*. Proc Natl Acad Sci USA..

[45] Hall AB, Papathanos P-A, Sharma A, Cheng C, Akbari OS, Assour L (2016). Radical remodeling of the Y chromosome in a recent radiation of malaria mosquitoes. Proc Natl Acad Sci USA..

[46] Hall AB, Qi Y, Timoshevskiy V, Sharakhova MV, Sharakhov IV, Tu Z (2013). Six novel Y chromosome genes in *Anopheles* mosquitoes discovered by independently sequencing males and females. BMC Genomics..

[47] Krzywinska E, Dennison NJ, Lycett GJ, Krzywinski J (2016). A maleness gene in the malaria mosquito *Anopheles gambiae*. Science..

[48] Criscione F, Qi Y, Tu Z. GUY1 confers complete female lethality and is a strong candidate for a male-determining factor in *Anopheles stephensi*. Elife. 2016;5 10.7554/eLife.19281. 10.7554/eLife.19281 PMC5061544 27644420

[49] Rose G, Krzywinska E, Kim J, Revuelta L, Ferretti L, Krzywinski J (2016). Dosage compensation in the African malaria mosquito *Anopheles gambiae*. Genome Biol Evol..

[50] Lycett GJ, Kafatos FC, Loukeris TG (2004). Conditional expression in the malaria mosquito *Anopheles stephensi* with Tet-On and Tet-Off systems. Genetics..

